# Pain neuroscience education: Which pain neuroscience education metaphor worked best?

**DOI:** 10.4102/sajp.v75i1.1329

**Published:** 2019-08-13

**Authors:** Adriaan Louw, Emilio J. Puentedura, Ina Diener, Kory J. Zimney, Terry Cox

**Affiliations:** 1 International Spine and Pain Institute, Story City, United States; 2 Department of Physical Therapy, Baylor University, Waco, United States; 3 Private, Stellenbosch, South Africa; 4 Department of Physical Therapy, University of South Dakota, Vermillion, United States; 5 Department of Physical Therapy, Southwest Baptist University, Bolivar, United States

**Keywords:** pain neuroscience education, metaphors, lumbar radiculopathy surgery, physiotherapy, survey, chronic pain

## Abstract

**Background:**

The use of pain neuroscience education (PNE) has been shown to be effective in reducing pain, improving function and lowering fear and catastrophisation. Pain neuroscience education utilises various stories and metaphors to help patients reconceptualise their pain experience. To date no individualised study has looked at which stories and metaphors may be the most effective in achieving the positive outcomes found with the use of PNE.

**Objectives:**

This study examined patient responses to the usefulness of the various stories and metaphors used during PNE for patients who underwent surgery for lumbar radiculopathy.

**Method:**

Twenty-seven participants who received preoperative PNE from a previous randomised control trial (RCT) were surveyed 1-year post-education utilising a 5-point Likert scale (0 – ‘do not remember’, 4 – ‘very helpful’) on the usefulness of the various stories and metaphors used during the PNE session. Participant demographics and outcomes data (pain intensity, function and pain knowledge) were utilised from the previous RCT for analysis and correlations.

**Results:**

Nineteen surveys were returned for a response rate of 70%. No story or metaphor mean was below 2 – ‘neutral’, lowest mean at 2.53; 6 of the 11 stories or metaphors scored a mean above 3 – ‘helpful’.

**Conclusion:**

No individual story or metaphor stood out as being predominately important in being helpful in the recovery process through the use of PNE.

**Clinical implications:**

The overall messages of reconceptualising pain during PNE may be more important than any individual story or metaphor.

## Introduction

Traditional biomedical education for pain-related musculoskeletal conditions focuses on a structural pathological model as a means of explaining why someone is going through a pain experience. There is evidence to show that biomedical education, which often produces potential negative expectations through verbal suggestions, may influence pain perception in a negative way (Blasini et al. 2017). Pain neuroscience education (PNE) is an educational strategy within the biopsychosocial model of care for individuals with pain-related musculoskeletal conditions. Pain neuroscience education incorporates the multidimensionality of a pain experience and helps patients reconceptualise pain through understanding the multiple neurophysiological, neurobiological, sociological and physical components that may be involved in their individual pain experience (Moseley 2007; Moseley & Butler 2015). Pain neuroscience education utilises various metaphors and analogies to explain the neurophysiological processes of pain occurring within the patient, along with the various other multidimensional aspects that may contribute to the patient’s pain experience.

Pain neuroscience education has shown promise as an effective educational strategy adjunct to a comprehensive multimodal rehabilitation programme (Louw et al. 2016). Various studies have shown, through the use of PNE, reductions in pain (Moseley 2002, 2003a), improved function (Van Oosterwijck et al. 2013; Vibe Fersum et al. 2013), decreased fear of movement (Téllez-García et al. 2014) and less catastrophising (Meeus et al. 2010).

While the effectiveness of the PNE approach has shown promise based on the overall concept of reconceptualising pain, the effectiveness of individual metaphors and analogies to accomplish this within a PNE session is less well known.

Even with the understanding that pain neurobiology and neurophysiology explained to patients via stories, metaphors and pictures are helpful, there is currently no evidence to determine if the total overall educational exposure (all of the stories) or single stories are most helpful in causing a conceptual shift in a person struggling with pain. Because of increasing demands on healthcare providers in regard to time management, resources distribution and healthcare costs (Orszag & Ellis 2007), it may be beneficial to explore this question, as a means of balancing cost-effectiveness of PNE delivery (Moseley 2003b). The aim of this study was to determine, from a patient’s perspective, which metaphors they found most helpful in developing a greater understanding of their pain experience 1 year after they had received a PNE session before lumbar surgery (LS) for radiculopathy.

## Research methods and design

### Design

Our study was a non-experimental cross-sectional descriptive survey of participants after PNE. It is one of the follow-up studies resulting from a multi-centre randomised controlled trial (RCT) of preoperative PNE before LS for radiculopathy, which has been published elsewhere (Louw et al. 2014).

### Participants

A total of 27 participants who completed the 1-year follow-up from the original RCT treatment group that received PNE were eligible for our study. Original inclusion criteria were the following: (1) scheduled for LS for radiculopathy; (2) willingness to comply with the predetermined follow-ups and (3) willingness to complete postoperative questionnaires at designated time intervals. Exclusion criteria were the following: (1) age younger than 18 years or older than 65 years; (2) not being proficient in reading or comprehending the English language; (3) scheduled for LS involving instrumentation (e.g. spinal fusion, arthroplasty); (4) participation in a formal back school or multidisciplinary pain management programme; (5) undergoing LS for a condition other than lumbar radiculopathy; (6) the presence of chronic-pain-related conditions (e.g. fibromyalgia, chronic fatigue syndrome) or (7) symptoms of cord compression.

### Preoperative pain neuroscience education

**FIGURE 1:**
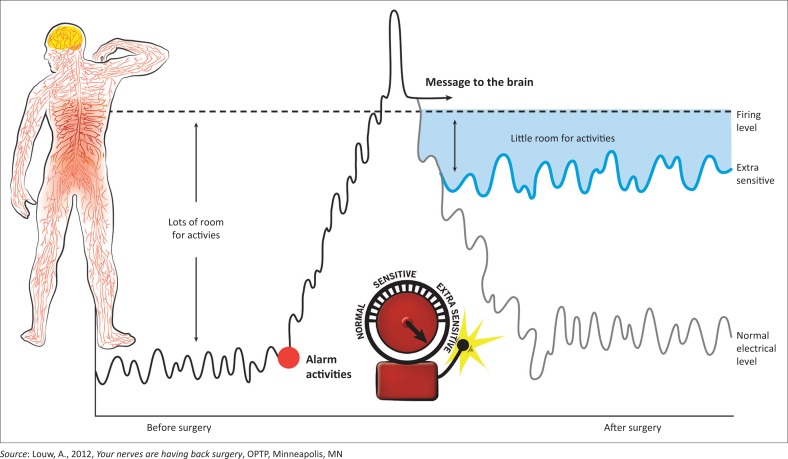
Example of picture to explain ‘extra-sensitive alarm’.

**FIGURE 2:**
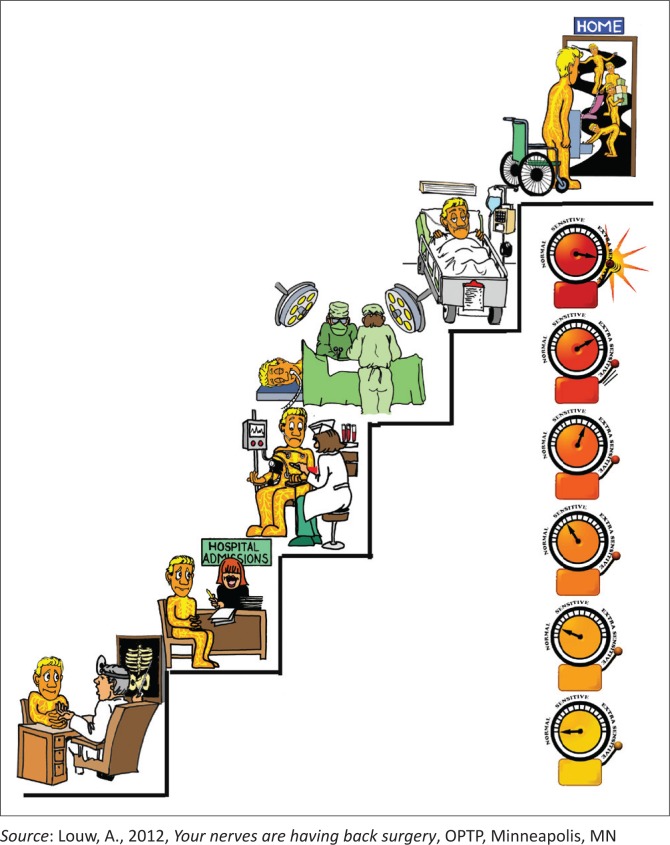
Example of picture to explain ‘hospital experiences’.

The development and content of the preoperative PNE session has been published elsewhere (Louw et al. 2013). Table 1 provides information about the content and the metaphors used, and Figures 1 and 2 demonstrate some of the pictures used with the metaphors to describe each aspect of the preoperative PNE programme. The PNE was provided by participating physiotherapists who had been trained and tested in the PNE programme. The PNE occurred in a one-on-one verbal format, with the use of pictures, examples, metaphors and drawings as needed. This was done in a conversational and personal approach rather than a lecture format. To ensure a standardised PNE programme, a systematic checklist was developed. The educational sessions averaged 30 min. Patients were additionally provided with a preoperative PNE booklet summarising the educational content of the preoperative PNE session, including pictures, examples and metaphors. Patients were asked to read the PNE booklet at least once before and once after their surgery.

**TABLE 1:** Description of the metaphors and target topics used in the preoperative pain neuroscience education.

Story	Metaphor	Target topic
1	Alarm system: Your nerves working like an alarm system to protect you	Neurons, synapses, action potential and nociception
2	Extra-sensitive alarm: The nerves (alarm system) in your back becoming extra sensitive	Peripheral sensitisation, neuropathic pain, central sensitisation and hyperalgesia
3	Nerve sensors: Nerve sensors telling you about movement, stress and cold	Ion channel expression, peripheral sensitisation, neuroplasticity and hyperalgesia
4	Yellow flags: Issues (yellow flags) that keep your alarm system extra sensitive	Biopsychosocial risk factors, fear avoidance and pain catastrophisation
5	Nosy neighbours: Why nerves can become sensitive and how spreading pain might occur	Neuroplasticity, hyperalgesia, peripheral sensitisation and immune responses
6	Hospital experiences: Surgery and hospital experiences ramping up the alarm system	Fear avoidance, pain catastrophisation and stress biology
7	Calming sensitive nerves: Calming down the alarm system – knowledge and movement	Cognitive therapy, inhibition, endogenous mechanisms of pain control, aerobic exercise, desensitisation and addressing fear
8	Hurt does not equal harm: Understanding ‘hurt does not equal harm’ and ‘sore but safe’ sayings about extra-sensitive nerves	Peripheral and central sensitisation, fear avoidance, coping strategies, behaviour change, goal setting, pacing and graded exposure
9	Dry and wet brain: The brain’s pain medicine	Endogenous mechanisms of pain control, neurotransmitters, inhibition and facilitation
10	No freaking over flare-ups: The ups and downs of normal recovery	Pacing, graded exposure, hyperalgesia, goal setting and internal locus of control
11	Pain is normal: Pain after surgery is to be expected and normal	Realistic goals, pain biologically normal, sensitisation and neuroplasticity

### Survey development

A survey was developed, based on the aims of the study and previous survey studies used by the research team in developing PNE programmes for low back pain and spine surgery (Louw et al. 2012, 2015b). The survey asked patients to recall 11 key elements of the PNE and indicate how helpful each story or example was in their recovery. The replies were scored using a Likert scale: 0 = do not remember, 1 = not very helpful, 2 = neutral, 3 = helpful and 4 = very helpful. In addition, each patient was asked to specifically write down which single story or example helped most, and there was space for general comments or feedback on the preoperative PNE information. To establish face and content validity, the draft survey was sent to a panel of 10 national and international experts in the field of PNE (Louw et al. 2012; Powell 2003). Experts were asked to provide feedback on the content and completion of the PNE survey and return comments in 3 weeks. A reminder email was sent to panelists if they had not completed the accompanying checklist for the survey after 3 weeks. The survey was deemed ready for the next phase when 70% agreement was obtained by the expert panel, following expert review, minor grammatical, punctuation and spacing changes were made. A pilot study comprising a convenience sample of five patients with chronic pain, from a clinic that specialises in treating chronic pain, was used to review the content. These patients had received similar PNE and were asked to review the content, the ease of completion and the time it took to complete the questionnaire (10 min). Upon further minor editing, the final survey was deemed ready for distribution.

### Survey distribution

After the 1-year follow-up for the RCT (Louw et al. 2014), each eligible participant was sent a letter, thanking them for their participation in the original RCT and asking them to complete the newly designed PNE metaphor survey. Participants were provided with a stamped, return addressed envelope for the completed survey. Participants who did not reply to the metaphor survey were contacted telephonically after 1 month and asked to please complete the survey.

### Data analysis

Demographic data and the outcomes of the survey data were extracted and entered into an Excel spreadsheet with conversion to SPSS version 24 (IBM Corporation) for full analysis. Descriptive statistics such as means, counts and percentages were used to describe the participants. Ranges, mode and frequency of each metaphor were calculated from survey data.

### Ethical considerations

The study was approved by the Health Research Ethics Committee at Stellenbosch University (Ethics Reference #: N09/09/247).

## Results

### Participants

Nineteen of the 27 participants (response rate = 70.3%) completed the PNE metaphor survey. Participants completing the survey had a mean age of 51.3 years and 11 were female (58%). Table 2 provides further demographic information of the participants completing the survey.

**TABLE 2:** Participant demographics *n* = 19.

Characteristics	*n*	%
Age, mean (s.d.), year	51.3	13.0
Female, *n* (%)	11	58
Race or ethnicity, *n* (%)		
White people	19	100
Education level, *n* (%)		
Postgraduate	6	32
College graduate	5	26
High school graduate	8	42
Primary reason for surgery, *n* (%)		
Pain	12	63
Numbness or paraesthesia	3	16
Decreased function	3	16
Failed treatment	1	5
Duration of back and leg pain prior to surgery, mean (s.d.), week	76.7	111.9

Data presented in number and percentage unless otherwise indicated.

s.d., standard deviation.

### Pain metaphors

Table 3 shows the range, mode, mean and standard deviations for each question as rated by each participant.

**TABLE 3:** Individual metaphor information.

Metaphor	Range	Mode	Mean	s.d.
Alarm system (*n* = 18)	2–4	3	3.11	0.58
Extra-sensitive alarm (*n* = 19)	2–4	3	3.16	0.77
Nerve sensors (*n* = 19)	2–4	3	3.00	0.67
Yellow flags (*n* =18)	0–4	3	2.78	1.00
Nosy neighbours (*n* = 18)	2–4	2	2.89	0.83
Hospital experiences (*n* = 19)	2–4	3	3.11	0.74
Calming sensitive nerves (*n* =18)	2–4	3	3.00	0.59
Hurt does not equal harm (*n* = 19)	1–4	3	2.84	0.69
Dry and wet brain (*n* =17)	0–4	2 and 3	2.53	0.94
No freaking over flare ups (*n* = 19)	2–4	3	2.79	0.63
Pain is normal (*n* = 19)	2–4	3	3.21	0.63

s.d., standard deviation.

**FIGURE 3:**
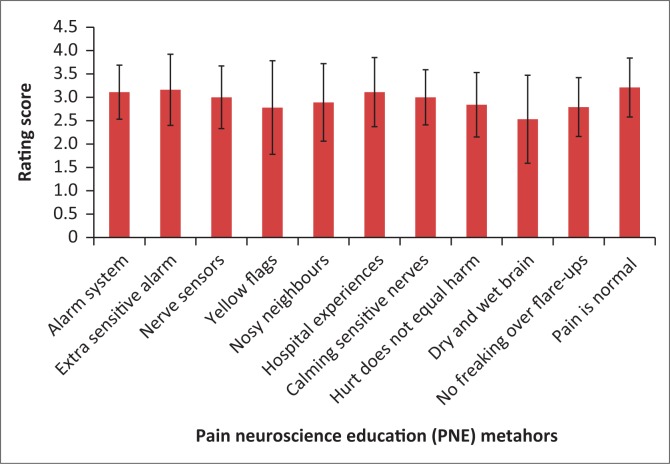
Rating of the pain neuroscience education metaphors (0 = cannot remember; 4 = very helpful).

The overall ranking (mean of the means) of the pain metaphors was 2.94 on the 0–4-point Likert scale. The highest ranking (mean score) metaphors in order of importance were (1) the general concept of pain being normal after surgery (3.21), (2) extra-sensitive alarm system (3.16), (3) the body’s living alarm system (3.11), (4) surgery experience influencing nerve sensitivity (3.11) and (5) nosy neighbours (2.89) (Figure 3).

One participant scored ‘dry and wet brain’ and ‘yellow flags’ as 0 – do not remember. Another participant scored ‘hurt does not equal harm’ as 1 – not very helpful. All of the other metaphor stories scored between 2 – neutral and 4 – very helpful on the Likert scale. The median score for all the metaphors was 3 – helpful. The mode for each metaphor ranked 3 – helpful, except for ‘nosy neighbours’ where the mode was 2 – neutral and ‘dry and wet brain’ which tied between 2 – neutral and 3 – helpful with the participants. The overall combined themes centred around two main issues: ‘easy to understand’ and ‘knew what to expect after surgery’.

## Discussion

This is the first study to investigate, from a patient’s perspective, if specific metaphors associated with PNE are more helpful than others. The results from this study indicate no one metaphor seems to be superior to other metaphors for people undergoing PNE prior to LS for radiculopathy at the 1-year recovery follow-up period. Patients are interested in learning more about pain (Louw, Louw & Crous 2009; Moseley 2003b). The results from this study show that patients value the metaphors and stories shared as part of the PNE. Only one participant had a ‘not helpful’ grading of the ‘hurt does not equal harm’ metaphor and another scored ‘dry and wet brain’ and ‘yellow flags’ as ‘do not remember’. The rest of the participants scored the metaphors between helpful and very helpful. These results, similar to earlier PNE research that shows patients are able to take on ‘complex’ pain issues, which are taught via metaphors, examples and images (Moseley 2003b). No single metaphor was found to be better than the others in our study. However, each individual story together seemingly created an overall story, which enabled the patient to develop a greater understanding of pain and their pain experience as a whole.

The layout of the material in our study was similar to the study by Gallagher et al., who had patients read a book of metaphors associated with pain science, covering 11 different sections, and not one specific story (Gallagher, Mcauley & Moseley 2013). The mentioned study showed that the combination of metaphors tested in the book provided a significant shift in patients’ pain catastrophisation and pain knowledge.

The PNE programme tested in this trial was a culmination of more than a half-dozen studies to develop the PNE programme, with the intent to address all (or most) of the pertinent information patients may need to know after undergoing lumbar radiculopathy surgery (Louw et al. 2009, 2012, 2013). The results would indicate the PNE programme attained this goal, given all the metaphors were ranked as important and no one metaphor was found to be most important.

From the patients’ perspective, the overall theme of ‘pain after surgery is expected and normal’ carried some weight, both in being ranked highest and in the summary of the general themes. This may be specific to this patient population as it has been shown that patients have a mismatch in regard to their expectations and experiences when it comes to LS (Toyone et al. 2005). Various studies have shown that patients expect to be ‘pain-free’ after LS, which may not be biologically plausible (Keulers et al. 2008; Mancuso et al. 2014; Toyone et al. 2005). It is argued that this mismatch may, indeed, increase the potential for persistent pain (Keulers et al. 2008; Louw et al. 2012). Louw et al. 2009 interviewed patients who underwent LS for radiculopathy 4 weeks after surgery and found 50% of the patients had increased fear that their pain would increase, rather than subside (Louw et al. 2009). Fear has been strongly correlated to increased pain experiences (Pincus et al. 2006; Poiraudeau et al. 2006; Simon, Stryker & George 2011; Vlaeyen & Linton 2000). In fact, a population-based survey of LS indicates that the general population expects a long recovery after LS (Landers et al. 2014). It is within this framework that preoperative PNE is so important to be applicable to the clinical situation and pain picture of the patient.

Pain is a normal human experience (Moseley 2007; Woolf 2000). It has been shown that pain is common following LS (Oosterhuis et al. 2014; Ostelo et al. 2003a, 2003b, 2004), and patients need to be prepared for this, understand it and develop healthy cognitions. In our study, for example, 60% of the patients underwent surgery because of a primary complaint of pain and when completing the survey 1 year after surgery, mean low back pain was still 4.1/10 (Numeric rating scale [NRS]). Traditional biomedical pain education model equates pain with injury, while PNE helps patients develop an understanding of a hypervigilant nervous system (Louw et al. 2013). Even though pain is experienced, it is reconceptualised as sensitisation versus injury. It is believed that this reconceptualisation then, in turn, normalises pain catastrophisation and fear avoidance, which supports the current evidence for PNE addressing these psychosocial issues (Gallagher et al. 2013; Louw et al. 2011).

All of the stories contained in the preoperative PNE programme centred around this theme of sensitisation and normality of pain, which further strengthens the argument why the sum of all the metaphors was seen as beneficial (Louw 2012), versus a single metaphor. In the development of the preoperative PNE programme, the authors conducted a case series of immediate changes of patients learning these metaphors and showed a more realistic expectation of pain after surgery (Louw, Diener & Puentedura 2015a), which agrees with the idea that it is the sum of all the metaphors.

While this is an initial exploratory survey investigating which PNE metaphors may be most effective and significant and follow-up research is needed, it may provide some clinical insights in the delivery of PNE. Our study showed that no one metaphor was more important than another. Therefore, clinicians do not have to feel tied into using a specific set protocol to deliver PNE. Considering the multidimensionality of pain and the heterogeneous nature of each individual in pain, this makes sense that the delivery of PNE in a clinical setting is probably best delivered in a heterogeneous fashion based on the patient in front of the clinician.

The most effective dosage (time spent) on NPE has not been established, and may vary hugely between acute and persistent pain situations, and between low and high sensitisation. The systematic review by Louw et al. (2011), and their follow-up systematic review 5 years later (Louw et al. 2016), did not inform our understanding as to which portions within the content of PNE might be the most effective and what time needs to be spent on PNE. Patients with low distress, and minimum psychosocial contributors to pain, may need a few specific metaphors. Given the complexity and multi-faceted aspects associated with persistent pain, including peripheral and central sensitisation, ion channel expression, fear avoidance, fear and anxiety, (Moseley 2007; Nijs et al. 2013), it is not surprising to find that a single or a few stories may in fact be inadequate to address all or most of the multidimensional aspects of pain that people may face. Reducing a multidimensional pain experience down to a few metaphors to describe and provide meaning to someone’s pain experience may not be adequate. A strong therapeutic alliance and clinical communication emphasising ‘listening’ to the patient (Diener, Kargela & Louw 2016) will enhance this process, as it may enable the therapist to use metaphors specifically applicable to the patient’s pain context. Another important clinical implication was that there appeared to be value in all of the PNE stories being tied together towards an overarching theme of why they had ongoing pain.

Therefore, which individual metaphors the clinician utilises may not be as important as long as they all tie into a central understanding of why the patient still hurts. As the patient triangulates information from the various stories and metaphors as part of the PNE process, it may be important that there are no contradictory messages from the healthcare provider and there is congruency in the message.

### Limitations

This study contains various limitations. The sample size is a small sample of convenience with no *a priori* determination of sample size, so it may be underpowered. The response rate of less than 100% leads to potential selection bias that may have occurred. It is also specific to patients who are recovering from lumbar radiculopathy surgery, all influencing the ability to generalise the findings of our study. The study design of selecting recall of the intervention at 1-year follow-up does not give an indication of metaphor preferences of participants earlier in the recovery process from surgery. Another limitation is the singularity of the mode and style of delivery within this specific study (Louw et al. 2014). While there are similarities and differences to the various PNE modes of delivery (Louw et al. 2016), no current exploratory study has looked at how the potential differences may affect outcomes differently. There is further research needed to explore how these differences may or may not affect outcomes, and until that is completed, caution should be taken in interpreting these results too broadly.

## Conclusion

No single metaphor or story was superior to other metaphors in the PNE process to help individuals in their recovery after lumbar radiculopathy surgery. Multiple pain metaphors, combined into one single story, may be important from a patient’s perspective, in helping them understand their pain experience.
